# Supplementary material for: ATM signaling modulates cohesin behavior in meiotic prophase and proliferating cells
Source: Nat Struct Mol Biol. 2023 Mar 6;30(4):436–50. doi: 10.1038/s41594-023-00929-5 (PMC10113158; doi:10.1038/s41594-023-00929-5)
Supplement: Supplementary file 1 — Supplementary text and references. [file 41594_2023_929_MOESM1_ESM.pdf]

nature structural & molecular biology

Article

https://doi.org/10.1038/s41594-023-00929-5

ATM signaling modulates cohesin behavior in meiotic prophase and proliferating cells

In the format provided by the authors and unedited

Supplementary Text

The activity of *C. elegans* ATL-1 is independent of phosphorylation at a CHK-2 motif

Like *C. elegans* ATM-1, ATL-1 also has a CHK-2 consensus at the C-terminus of its FAT domain (**Extended Data Fig. 3a**). To test whether this CHK-2 consensus motif plays a similar role in regulating ATL-1, we constructed a nonphosphorylatable mutant (*atl-1^{KA}*) and assayed the embryonic viability and meiotic nondisjunction of homozygous mutant animals[1]. As previously reported, the *atl-1* null allele *tm853* showed 100% embryonic lethality (**Extended Data Fig. 3b**)[2]. By contrast, animals homozygous for the *atl-1^{KA}* allele showed normal viability and fertility, indicating that this motif is dispensable for ATL-1 activity (**Extended Data Fig. 3b**).

WAPL depletion leads to the formation of “vermicelli” in the absence of ATM

Our results indicate that ATM downregulates WAPL to promote cohesin enrichment at DNA damage foci. We therefore wondered whether depletion of WAPL might restore RAD21 foci upon DNA damage in the absence of ATM activity. However, WAPL knockdown resulted in the appearance of “vermicelli” even in the absence of ATM (**Extended Data Fig. 7i-k**), likely reflecting global stabilization of cohesin. Under these conditions, we did not detect any specific enrichment of cohesin at damage foci.

References

1. Hodgkin, J., Horvitz, H. R. & Brenner, S. NONDISJUNCTION MUTANTS OF THE NEMATODE CAENORHABDITIS ELEGANS. *Genetics* **91**, 67–94 (1979).
2. Garcia-Muse, T. & Boulton, S. J. Distinct modes of ATR activation after replication stress and DNA double-strand breaks in Caenorhabditis elegans. *The EMBO Journal* **24**, 4345–4355 (2005).
