# Supplementary material for: ATM signaling modulates cohesin behavior in meiotic prophase and proliferating cells
Source: Nat Struct Mol Biol. 2023 Mar 6;30(4):436–50. doi: 10.1038/s41594-023-00929-5 (PMC10113158; doi:10.1038/s41594-023-00929-5)
Supplement: Supplementary file 2 — Reporting Summary [file 41594_2023_929_MOESM2_ESM.pdf]

nature portfolio

Corresponding author(s): Abby F. Dernburg

Last updated by author(s): Nov 15, 2022

Reporting Summary

Nature Portfolio wishes to improve the reproducibility of the work that we publish. This form provides structure for consistency and transparency in reporting. For further information on Nature Portfolio policies, see our Editorial Policies and the Editorial Policy Checklist.

Statistics

For all statistical analyses, confirm that the following items are present in the figure legend, table legend, main text, or Methods section.

n/a	Confirmed	
☐	☒	The exact sample size (*n*) for each experimental group/condition, given as a discrete number and unit of measurement
☐	☒	A statement on whether measurements were taken from distinct samples or whether the same sample was measured repeatedly
☐	☒	The statistical test(s) used AND whether they are one- or two-sided<br>*Only common tests should be described solely by name; describe more complex techniques in the Methods section.*
☒	☐	A description of all covariates tested
☐	☒	A description of any assumptions or corrections, such as tests of normality and adjustment for multiple comparisons
☐	☒	A full description of the statistical parameters including central tendency (e.g. means) or other basic estimates (e.g. regression coefficient) AND variation (e.g. standard deviation) or associated estimates of uncertainty (e.g. confidence intervals)
☐	☒	For null hypothesis testing, the test statistic (e.g. *F*, *t*, *r*) with confidence intervals, effect sizes, degrees of freedom and *P* value noted<br>*Give P values as exact values whenever suitable.*
☒	☐	For Bayesian analysis, information on the choice of priors and Markov chain Monte Carlo settings
☒	☐	For hierarchical and complex designs, identification of the appropriate level for tests and full reporting of outcomes
☒	☐	Estimates of effect sizes (e.g. Cohen's *d*, Pearson's *r*), indicating how they were calculated

Our web collection on statistics for biologists contains articles on many of the points above.

Software and code

Policy information about availability of computer code

Data collection	DeltaVision/softWoRx
Data analysis	ImageJ/Fiji (v1.53t), scikit-image library v0.18 (Python 3.9) and ImageJ macros and Python scripts to automate the image analysis tasks (https://github/zhouliangyu). Microscopy image blending is processed in Adobe Photoshop CC 2017.

For manuscripts utilizing custom algorithms or software that are central to the research but not yet described in published literature, software must be made available to editors and reviewers. We strongly encourage code deposition in a community repository (e.g. GitHub). See the Nature Portfolio guidelines for submitting code & software for further information.

Data

Policy information about availability of data

All manuscripts must include a data availability statement. This statement should provide the following information, where applicable:

- Accession codes, unique identifiers, or web links for publicly available datasets
- A description of any restrictions on data availability
- For clinical datasets or third party data, please ensure that the statement adheres to our policy

Source data are provided with this paper.

Human research participants

Policy information about studies involving human research participants and Sex and Gender in Research.

Reporting on sex and gender	*Use the terms sex (biological attribute) and gender (shaped by social and cultural circumstances) carefully in order to avoid confusing both terms. Indicate if findings apply to only one sex or gender; describe whether sex and gender were considered in study design whether sex and/or gender was determined based on self-reporting or assigned and methods used. Provide in the source data disaggregated sex and gender data where this information has been collected, and consent has been obtained for sharing of individual-level data; provide overall numbers in this Reporting Summary. Please state if this information has not been collected. Report sex- and gender-based analyses where performed, justify reasons for lack of sex- and gender-based analysis.*
Population characteristics	*Describe the covariate-relevant population characteristics of the human research participants (e.g. age, genotypic information, past and current diagnosis and treatment categories). If you filled out the behavioural & social sciences study design questions and have nothing to add here, write "See above."*
Recruitment	*Describe how participants were recruited. Outline any potential self-selection bias or other biases that may be present and how these are likely to impact results.*
Ethics oversight	*Identify the organization(s) that approved the study protocol.*

Note that full information on the approval of the study protocol must also be provided in the manuscript.

Field-specific reporting

Please select the one below that is the best fit for your research. If you are not sure, read the appropriate sections before making your selection.

☒ Life sciences ☐ Behavioural & social sciences ☐ Ecological, evolutionary & environmental sciences

For a reference copy of the document with all sections, see nature.com/documents/nr-reporting-summary-flat.pdf

Life sciences study design

All studies must disclose on these points even when the disclosure is negative.

Sample size	We quantified at least 25 cells for each mutant/group/condition in every assay, because a sample that has a size of >25 is generally considered to be large enough to reflect if the data is distributed normally. In most cases in the study, the numbers (sample size) ranged from several tens to above a hundred.
Data exclusions	We quantified all intact cells that are not close to the edges of any image we collected. For C. elegans germline images, we only quantified meiotic cells that are in the stage of meiotic onset/early meiosis (within 10-20 rows of cells after the transition from mitosis to meiosis). For mammalian cell culture images we excluded mitotic cells and only quantified interphase cells. This is because cohesin-enriched damage foci are only visible in interphase nuclei.
Replication	For data that were collected from each mutant/group/condition, we quantified meiotic cells of at least three gonads from individual worms. All replicate experiments are successful and meaningful. For DNA damage foci analysis, we analyzed cells from at least three biological replicates. From all replicates the results we obtained are similar.
Randomization	Worms freely crawl on plates and we picked worms from each mutant/group/condition at random. When acquiring images, of either C. elegans gonads or mammalian cell cultures, we picked gonads/fields of cells at random.
Blinding	Complete blinding is not applicable, because the person who carried out the experiments knew the genetic backgrounds/conditions of worms/cells from each group before image acquisition. However, when analyzing the images we acquired, we apply the same criteria/scripts on the raw data across all conditions to minimize human intervention.

Reporting for specific materials, systems and methods

We require information from authors about some types of materials, experimental systems and methods used in many studies. Here, indicate whether each material, system or method listed is relevant to your study. If you are not sure if a list item applies to your research, read the appropriate section before selecting a response.

Materials & experimental systems

n/a	Involved in the study
☐	☒ Antibodies
☐	☒ Eukaryotic cell lines
☒	☐ Palaeontology and archaeology
☐	☒ Animals and other organisms
☒	☐ Clinical data
☒	☐ Dual use research of concern

Methods

n/a	Involved in the study
☒	☐ ChIP-seq
☒	☐ Flow cytometry
☒	☐ MRI-based neuroimaging

Antibodies

Antibodies used	Primary antibodies were purchased from commercial sources or have been described in previous studies, and were diluted as follows: rabbit anti-RAD-51 (Harper 2011, 1:500), rabbit anti-pHIM-8/ZIMs (Kim 2015, 1:500), goat anti-SYP-1 (Harper 2011, 1:300), chicken anti-HTP-3 (MacQueen 2005, 1:500), mouse anti-HA (Thermo Fisher 26183, 1:400), mouse anti-FLAG (Sigma F3165, 1:500), mouse anti-V5 (Thermo Fisher R960-25, 1:500), rabbit anti-V5 (Millipore Sigma V8137, 1:250), mouse anti-WAPL (Santa Cruz sc-365189, 1:500), rabbit anti-γH2A.X antibody (Cell Signaling, Cat No. 2577, 1:500), mouse anti-ATM antibody (Thermo Fisher, Cat No. MA1-23152, 1:500), rabbit anti-pS/TQ antibody (Cell Signaling, Cat No. 6966, 1:500), rabbit anti-COH-3/4 antibody (SDQ3972, ModENCODE project (Gerstein 2010), 1:500), rabbit anti-REC-8 antibody (SDQ0802, ModENCODE project (Gerstein 2010), 1:500), rabbit anti-WAPL-1 antibody (SDQ3963, ModENCODE project (Gerstein 2010), 1:500). Secondary antibodies raised in donkey and labeled with Alexa 488, Cy3, or Cy5 (Jackson ImmunoResearch Laboratories, 1:400, Alexa Fluor 488 Donkey anti-mouse #715-545-151, Alexa Fluor 488 Donkey anti-chicken #703-545-155, Alexa Fluor 488 Donkey anti-goat #705-545-147, Cy3 Donkey anti-mouse #715-165-151, Cy3 Donkey anti-rabbit #711-165-152, Cy3 Donkey anti-chicken #703-165-155, Cy5 Donkey anti-mouse #715-175-151, Cy5 Donkey anti-chicken #703-175-155, Alexa Fluor 647 Donkey anti-mouse #715-605-151, Alexa Fluor 647 Donkey anti-goat #705-605-147, Alexa Fluor 647 Donkey anti-rabbit #711-605-152).
Validation	Antibodies that are commercially available were validated by the manufacturers: Thermo anti-HA #26183 was validated by IP, IF, ICC, WB using mammalian cells/tissues and bacteria lysates. Sigma anti-FLAG F3165 was validated by IB, IP, ICC, IF, ELISA, ChIP, EIA, electron microscopy, flow cytometry. Thermo anti-V5 R960-25 was validated by WB, IHC, ICC, IF, ELISA, IP, ChIP, RIP and flow cytometry. Sigma anti-V5 V8137 was validated by IF, ICC, IP and WB. Santa Cruz anti-WAPL sc-365189 was validated by WB, IP, IF, ELISA, using human cultured cells. Cell signaling anti-gammaH2A.X antibody was validated by IF, WB and flow cytometry using mammalian cultured cells. Thermo anti-ATM #MA1-23152 was validated by IHC, WB using human cultured cells/tissues. Cell signaling anti-pS/TQ #6966 was validated by IP, WB, IHC, ChIP, IF using mammalian cultured cells and claimed to cross-react with C. elegans. Other antibodies were validated in previous studies (see above).

Eukaryotic cell lines

Policy information about cell lines and Sex and Gender in Research

Cell line source(s)	No new mammalian cell lines were generated in the study. The HeLa cells used in the study were obtained from UC Berkeley cell culture facility.
Authentication	No formal cell line authentication was carried out by the authors, only visual examination of the cell identity were performed every day during the cells were in use. Regular verification of the identities of cell lines was performed in the cell culture facility.
Mycoplasma contamination	Tested negative before being used in the study.
Commonly misidentified lines (See ICLAC register)	No misidentified lines were used in the study.

Animals and other research organisms

Policy information about studies involving animals; ARRIVE guidelines recommended for reporting animal research, and Sex and Gender in Research

Laboratory animals	This study utilized the invertebrate model organism C. elegans. Details of the genetic backgrounds of all alleles used in this study were listed in Source Data Table 1 in the manuscript.
Wild animals	No wild animals used in the study.
Reporting on sex	C. elegans can develop as one of two sexes, male or hermaphrodite, the latter being the major form of the organism.We analyzed C. elegans hermaphrodites (young adults) as a general practice for meiosis studies using this organism unless addressing male-specific questions.
Field-collected samples	No field-collected samples in the study.
Ethics oversight	C. elegans as a non-vertebrate model organism has no ethical restrictions.

Note that full information on the approval of the study protocol must also be provided in the manuscript.
