# Supplementary figures and images for: ATM signaling modulates cohesin behavior in meiotic prophase and proliferating cells
Source: Nat Struct Mol Biol. 2023 Mar 6;30(4):436–50. doi: 10.1038/s41594-023-00929-5 (PMC10113158; doi:10.1038/s41594-023-00929-5)

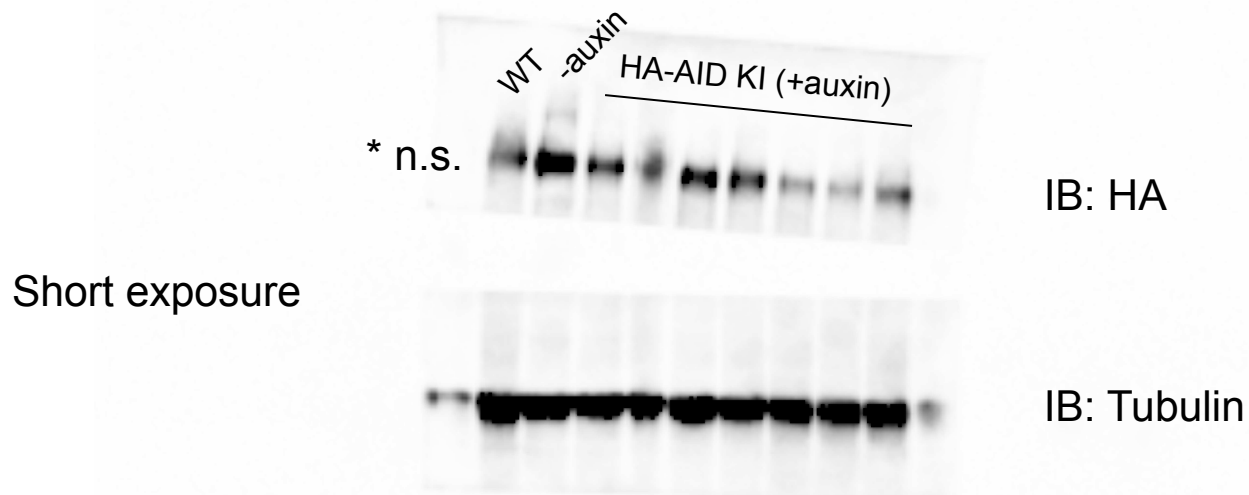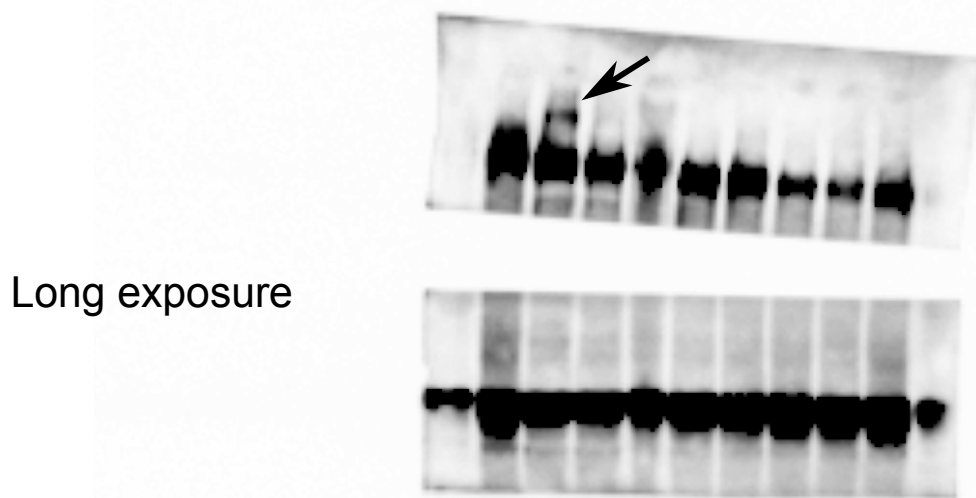

Unprocessed Western blot images for Extended Data Figure 2g

Supplement: Source Data Extended Data Fig. 2 — Uncropped western blot images. [file 41594_2023_929_MOESM17_ESM.pdf]
